# Application of computer‐aided detection for NCCN‐based follow‐up recommendation in subsolid nodules: Effect on inter‐observer agreement

**DOI:** 10.1002/cam4.6967

**Published:** 2024-02-01

**Authors:** Wu Quanyang, Zhou Lina, Huang Yao, Wang Jiawei, Tang Wei, Qi Linlin, Zhang Zewei, Hou Donghui, Li Hongjia, Chen Shuluan, Zhang Jiaxing, Zhao Shijun

**Affiliations:** ^1^ Department of Diagnostic Radiology, National Cancer Center/National Clinical Research Center for Cancer/Cancer Hospital Chinese Academy of Medical Sciences and Peking Union Medical College Beijing China; ^2^ PET‐CT Center, National Cancer Center/National Clinical Research Center for Cancer/Cancer Hospital Chinese Academy of Medical Sciences and Peking Union Medical College Beijing China

**Keywords:** computer‐aided diagnosis, low‐dose computed tomography, lung cancer, observer variation, subsolid nodule

## Abstract

**Rationale and Objectives:**

Computer‐aided detection (CAD) of pulmonary nodules reduces the impact of observer variability, improving the reliability and reproducibility of nodule assessments in clinical practice. Therefore, this study aimed to assess the impact of CAD on inter‐observer agreement in the follow‐up management of subsolid nodules.

**Materials and Methods:**

A dataset comprising 60 subsolid nodule cases was constructed based on the National Cancer Center lung cancer screening data. Five observers independently assessed all low‐dose computed tomography scans and assigned follow‐up management strategies to each case according to the National Comprehensive Cancer Network (NCCN) guidelines, using both manual measurements and CAD assistance. The linearly weighted Cohen’s kappa test was used to measure agreement between paired observers. Agreement among multiple observers was evaluated using the Fleiss kappa statistic.

**Results:**

The agreement of the five observers for NCCN follow‐up management categorization was moderate when measured manually, with a Fleiss kappa score of 0.437. Utilizing CAD led to a notable enhancement in agreement, achieving a substantial consensus with a Fleiss kappa value of 0.623. After using CAD, the proportion of major and substantial management discrepancies decreased from 27.5% to 15.8% and 4.8% to 1.5%, respectively (*p* < 0.01). In 23 lung cancer cases presenting as part‐solid nodules, CAD significantly elevates the average sensitivity in detecting lung cancer cases presenting as part‐solid nodules (overall sensitivity, 82.6% vs. 92.2%; *p* < 0.05).

**Conclusion:**

The application of CAD significantly improves inter‐observer agreement in the follow‐up management strategy for subsolid nodules. It also demonstrates the potential to reduce substantial management discrepancies and increase detection sensitivity in lung cancer cases presenting as part‐solid nodules.

## 1 INTRODUCTION

Low‐dose computed tomography (LDCT) screening can detect nodules. Pulmonary nodules are clinically significant since they may be early manifestations of lung cancer. Therefore, the accurate assessment and timely follow‐up of these nodules are crucial. However, explaining LDCT lung cancer screening results and formulating accurate follow‐up recommendations are labor‐intensive for radiologists.^1^ Therefore, to minimize the uncertainty and variability in the evaluation and management of pulmonary nodules, as well as standardize the reporting of LDCT screening results, the National Comprehensive Cancer Network (NCCN) in the United States developed a lung cancer screening classification and scoring system to assist radiologists in providing the most appropriate follow‐up recommendations.^2^

Numerous studies have extensively explored the progression and development of pulmonary nodules detected using CT in various lung cancer screening programs.^3^, ^4^, ^5^ Subgroup analyses based on nodule classification have revealed different malignant tendencies of subsolid nodules at different time points. Many persistent subsolid nodules represent early‐stage invasive adenocarcinoma.^6^ Furthermore, persistent non‐solid nodules gradually evolve into part‐solid nodules, with reports indicating that approximately 9.0% of these progressing to minimally invasive or invasive adenocarcinomas.^7^, ^8^ However, in the Asian region, due to differences in epidemiological profiles, healthcare systems, and accessibility of technology, the standards for lung cancer screening and characteristics of nodules may vary significantly compared to the Western countries. These variations can affect the detection rates of nodules, the types of nodules predominantly found, and the subsequent management strategies adopted. The results of a multicenter study on baseline LDCT lung cancer screening conducted in Shanghai indicate that the proportions of nonsolid nodules, part‐solid nodules, and solid nodules among lung cancer patients were 52.94%, 31.93%, and 15.13%, respectively.^9^ A study on LDCT screening targeting different lung cancer risk groups in Taiwan revealed that the proportion of subsolid nodules among participants with a family history of lung cancer was significantly higher than that in participants without such history (17.7% vs. 5.2%).^10^ Consequently, subsolid nodules in the Asian population warrant more focused attention. In contrast, overdiagnosis can lead to anxiety and unnecessary treatment. Therefore, reasonable and consistent follow‐up recommendations are crucial for the effective management of nodules.

Pivotal factors in the management of subsolid nodules include the presence of any solid component and their dimensions. However, visual assessment of nodule types and manual diameter measurements is influenced by significant inter‐observer variability, posing challenges to the accurate characterization and measurement of nodules, leading to inconsistent interpretations and management decisions.^11^, ^12^ Previous studies have shown varying degrees of agreement among observers in distinguishing solid and subsolid nodules and in assigning Lung‐RADS categories.^13^

The reliance on subjective assessments emphasizes the need for standardized and objective approaches to evaluate nodules, such as adopting advanced imaging techniques or implementing computer‐aided detection (CAD) systems. CAD systems not only enhance the sensitivity of nodule detection but also enable further nodule volume measurement, automatic segmentation, classification, and risk assessment.^14^, ^15^ Thus, incorporating CAD with human observers for double reading may help mitigate the impact of observer variability and improve the reliability and reproducibility of nodule assessments in clinical practice.^16^, ^17^ Therefore, this study aimed to evaluate the inter‐observer agreement of follow‐up recommendations using CAD for subsolid nodules.

## 2 MATERIALS AND METHODS

### 2.1 NCCN management classification and study groups

Nodules in the dataset used in this study were categorized according to the NCCN guidelines (Version 2023.01, Data S1).^2^ The observers recorded the type, size, and location of each dominant‐risk nodule. The NCCN guidelines inform nodule type identification (solid, part‐solid, or nonsolid) and size measurement (the longest diameter and its perpendicular diameter in the axial view/2). Recommendations for subsequent follow‐up were determined by the presence of risk‐dominant nodules. There were four recommendation categories: repeat scans at 0, 3, 6, and 12 months, in this context, a “0‐month follow‐up” refers to immediate subsequent evaluations within a short timeframe, including positron emission tomography and computed tomography (PET‐CT) scans and biopsy.

### 2.2 Data collection

**FIGURE 1:**
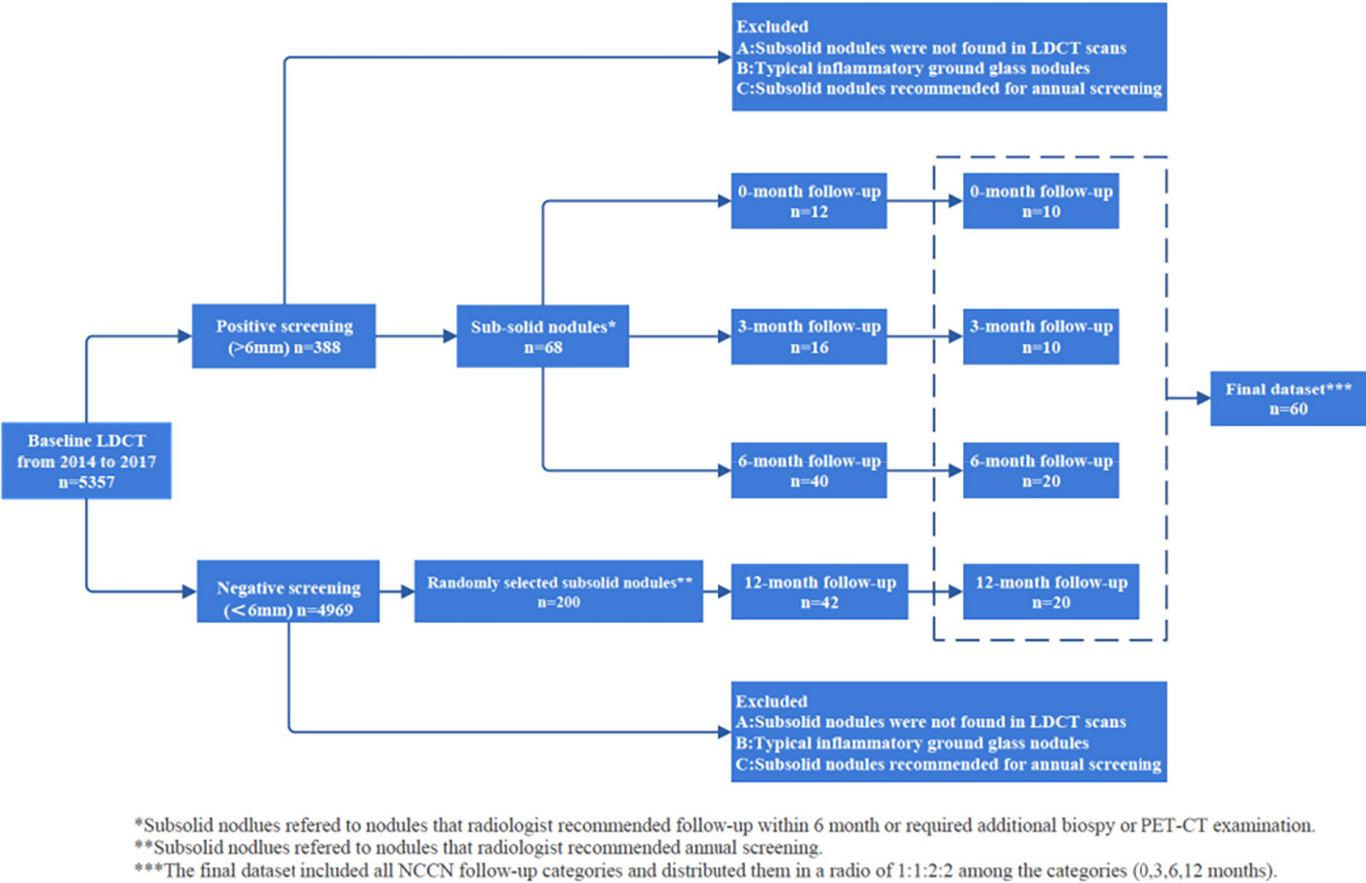
Flowchart for construction of subsolid nodule dataset.

Between 2014 and 2017, we obtained LDCT scans and essential patient details from individuals who underwent lung cancer screening at the National Cancer Center. The screening results for all patients were categorized in the system into positive (>6 mm), indeterminate (4–6 mm), and negative (<4 mm) nodules. In the first step, we analyzed radiological reports from individuals with positive nodules that included specific terms related to subsolid nodules, such as ground‐glass, nonsolid, subsolid, and part‐solid nodules. At this stage, our study incorporated subsolid nodules from reports recommending additional evaluation within 6 months or those recommending biopsy and PET‐CT scan. After excluding the descriptions of typical ground‐glass opacities associated with inflammation, 68 patients with subsolid nodules were included in this step. In the second step, we randomly chose 200 LDCT reports of participants with semi‐positive nodules, all of which recommended annual repeat screening. Concurrently, 42 LDCT cases with subsolid nodules were included. In the third step, two experienced radiologists (with 20 and 15 years of chest expertise, respectively) individually assessed the initial 110 (68 + 42) cases, aligning each with a management strategy based on the NCCN guidelines to establish a benchmark for subsequent management. Discrepancies were resolved by a chief radiologist with 30 years of chest expertise. Ultimately, the count at 0‐, 3‐, 6‐, and 12‐month follow‐ups were 12, 16, 40, and 42, respectively. In the fourth step, to maintain a balanced representation across all follow‐up categories, we created a dataset encapsulating every NCCN follow‐up classification proportioned in a 1:1:2:2 ratio across intervals (0, 3, 6, and 12 months). As a result, the final dataset for interobserver agreement assessment consisted of 60 scans from 60 participants, and 29 patients were pathologically confirmed to have lung cancer. Figure 1 presents a detailed flowchart of the dataset composition.

The Ethics Committee of Cancer Hospital, Chinese Academy of Medical Sciences approved this retrospective study and waived the need for written informed consent.

### 2.3 LDCT scan parameters

CT scans were conducted using 64‐detector row scanners from various manufacturers, including the General Electric Medical Systems (Discovery CT750 HD, or Optima CT660) and the Siemens Medical Systems (MOMATOM go Top or Definition Edge). Scans were performed at full inspiration, following standardized parameters, including a tube voltage of 120 kVp, automatic current time ranging from 25 to 100 mA with a rotation time of 0.5 s, and a slice thickness of 5 mm. Reconstructed images were generated using a standard algorithm with thicknesses of 1.0 or 1.25 mm and an interval of 0.8 mm.

### 2.4 CAD for automatic nodule classification

**FIGURE 2:**
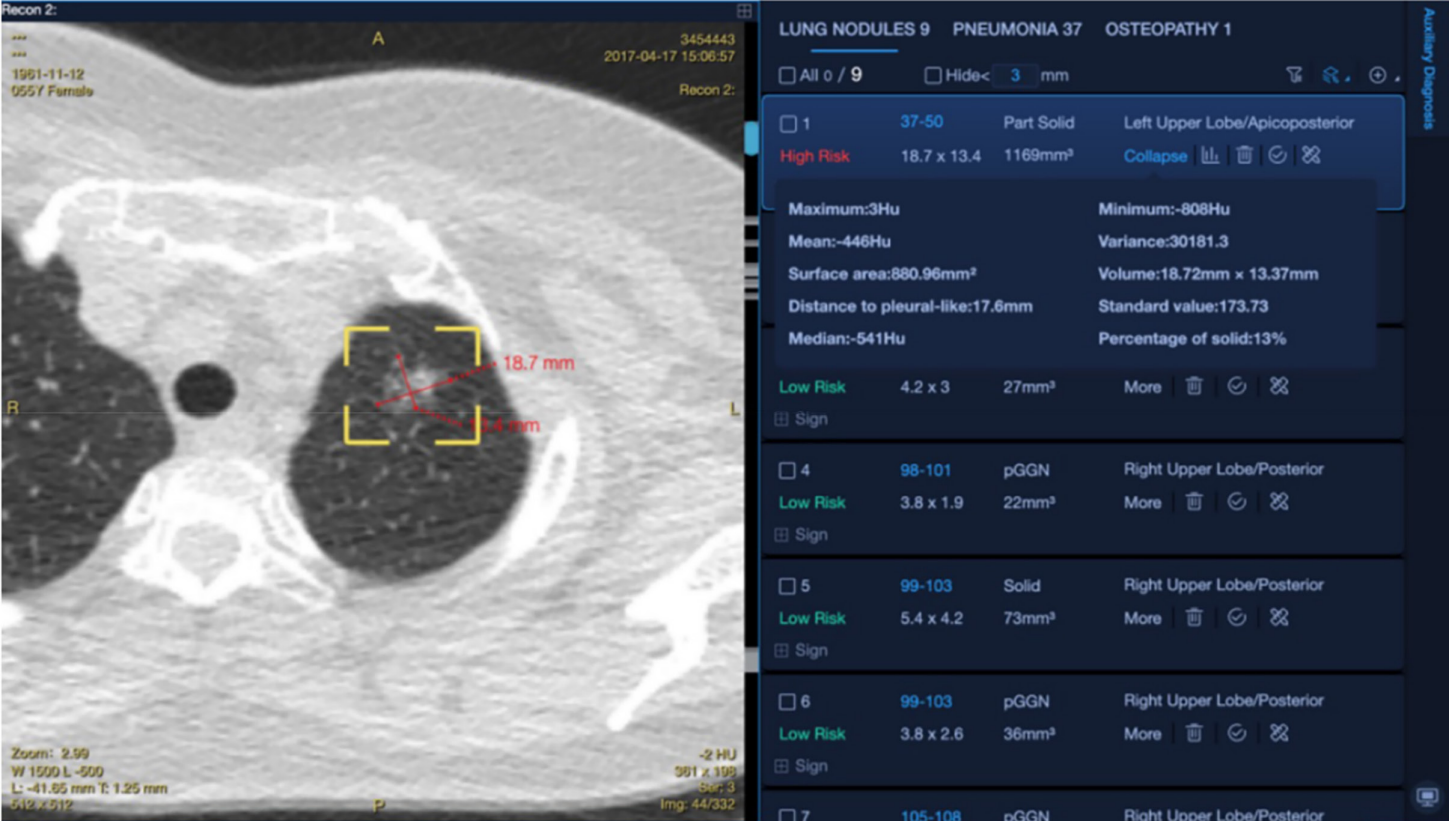
Nodule detection interface of the CAD software.

A deep learning‐based CAD system (Medical Imaging Assisted Diagnosis Platform, v.3.5.2; Huiying Medical Technology, Beijing) was utilized for the automatic classification of nodules in LDCT scans. After identifying the nodules, conducting a segmentation‐based assessment and size evaluation, all pinpointed nodules (with a threshold >3 mm) were prioritized based on their risk level. The CAD‐generated list of nodules was displayed in a separate window, as shown in the illustration. This list provides information about the nodules detected using CAD, including their location, nodule type, two‐dimensional diameter, volume, volume of solid components in partially solid nodules, and nodule risk level (Figure 2).

### 2.5 Observer and reading method

In this study, each observer performed two assessment rounds for all LDCT scans. The first round was performed manually and independently, whereas the second round was conducted with the assistance of CAD. The two rounds of assessment were spaced 2 weeks apart to eliminate recall bias. Five observers participated in this study, including two chest radiologists with 5 and 20 years of experience in chest diagnostics. The remaining three observers were radiology residents. The types of nodules are classified as part‐solid nodules, nonsolid nodules, and solid nodules. All lung cancer cases have been histologically confirmed. In the first round, observers recorded the type, size, and location of the risk‐dominant nodule and provided follow‐up recommendations for each case. Determination of the nodule type (solid, part‐solid, or nonsolid) and size (calculated as the average of the longest and perpendicular diameters in the axial plane) relied on annotations provided in the NCCN guidelines. All measurements were conducted using images with layer thicknesses of 1 mm or 1.25 mm. According to the NCCN guidelines, observers were required to classify the follow‐up recommendations into four categories (0, 3, 6, and 12 months) based on the level of risk associated with the nodules. Throughout the evaluation phase, all the observers were familiar with and referred to the printed version of the NCCN guidelines.

During the second round, the observers analyzed the CT images in a simultaneous‐observation setting, considering the annotations produced by the CAD. Upon evaluation of the entire LDCT scan, the observers were given the choice to identify a nodule detected by CAD or any newly discovered nodule as the risk‐dominant nodule. In certain scenarios, such as when the demarcation between a nodule and surrounding blood vessels is unclear, if the observers found the nodule type or size of the CAD‐detected nodules to be incorrect, they could modify the nodule type and measurement values. In such cases, the software automatically saved the modified results.

### 2.6 Statistics analysis

Inter‐rater agreement among multiple observers was evaluated using the Fleiss' kappa test. The linearly weighted Cohen's kappa test was used to measure agreement between paired observers. The interpretation of kappa values adhered to the Landis and Koch criteria.^18^ Discordant follow‐up management categories between the observers were analyzed based on differences in risk‐dominant nodule selection, nodule type, and measurement. McNemar's test was used to compare the proportions of these differences between the two rounds in the overall reading pairs. For each observer, changes in the follow‐up recommendation category between the two reading rounds were assessed.

To evaluate the effect of inconsistent classifications on patient management, inconsistent cases were classified into two categories based on variations in follow‐up recommendations. Minor inconsistency referred to a discrepancy shorter than 6 months (such as a 6‐month follow‐up vs. a 3‐month follow‐up), whereas major inconsistency was of 6 months or more (such as a 12‐month follow‐up vs. a 6‐month follow‐up; a 6‐month follow‐up vs. a 0‐month follow‐up). Furthermore, a substantial discrepancy was defined as a variation of at least 9 months in follow‐up time, specifically arising from the 12‐month versus 0/3‐month follow‐up recommendations.

The sensitivity of both the manual measurement and CAD in classicification of lung cancer was determined using a 6‐month follow‐up recommendation as a positive threshold. The sensitivities of the combined observers across the two rounds of reading were compared using generalized estimating equations. *p* < 0.05 was deemed statistically significant, and the Statistical Package for the Social Sciences (SPSS, version 27) was used for statistical calculations.

## 3 RESULTS

### 3.1 Demographics results

The median age of the participants was 54 years, with 30 men (50.0%) and 30 women (50.0%). Of them, 38.3% were either current or former smokers. Most of the participants had a history of passive smoking (75.0%), and 21.7% had a family history of lung cancer, and 36.7% of other malignant tumors. Of the 60 patients, 29 were diagnosed with lung cancer, and all cases of lung cancer were pathologically confirmed as adenocarcinoma. Furthermore, all diagnosed instances were categorized as Stage I lung cancer. Table 1 presents the detailed participant demographic information.

**TABLE 1:** Summarizes the basic demographic information of 60 participants.

Subject characteristics	Value (%)
Age (year)^a^	54 (40–81)
40–49	10 (16.7)
50–59	35 (58.3)
60–69	13 (21.7)
≥70	2 (3.3)
Sex	
Male	30 (50.0)
Female	30 (50.0)
Smoking status	
Current/Former smoker	23 (38.3)
No smoker	31 (51.7)
Unknown	6 (10.0)
Passive smoking	
No	9 (15.0)
Yes	45 (75.0)
Unknown	6 (10.0)
Education	
High school diploma or below	16 (26.7)
Associate's degree	11 (18.3)
Bachelor's degree	20 (33.3)
Master's degree or above	5 (8.3)
Unknown	8 (13.3)
Family history of respiratory disease	
COPD	0 (0)
Asthma	0 (0)
Other respiratory disease	44 (73.3)
Family history of cancer	
Lung cancer	13 (21.7)
Other malignancies	22 (36.7)
Asbestos exposure or occupational exposure	
No	52 (86.7)
Yes	2 (3.3)
Unknown	6 (10.0)
Diabetes	
No	50 (83.3)
Yes	2 (3.3)
Unknown	8 (13.3)
Screening results	
Lung cancer	29
Undetermined	31

^a^ Data are median value and numbers in parentheses are the range.

### 3.2 Follow‐up category agreement: Observers without CAD

Moderate agreement was achieved among the five observers, with a Fleiss kappa value of 0.437 (0.388–0.487). The Fleiss kappa value for the three radiology residents was 0.350 (0.260–0.440). The weighted Cohen kappa values among the paired observers ranged between 0.419 (0.264–0.574) and 0.777 (0.665–0.889), with an average Cohen kappa value of 0.609 (0.518–0.701) for all paired observers. The chest radiologists achieved substantial agreement with a Cohen's kappa value of 0.655 (0.503–0.807). Among radiology residents, the average weighted kappa value was 0.533 (0.501–0.565).

**FIGURE 3:**
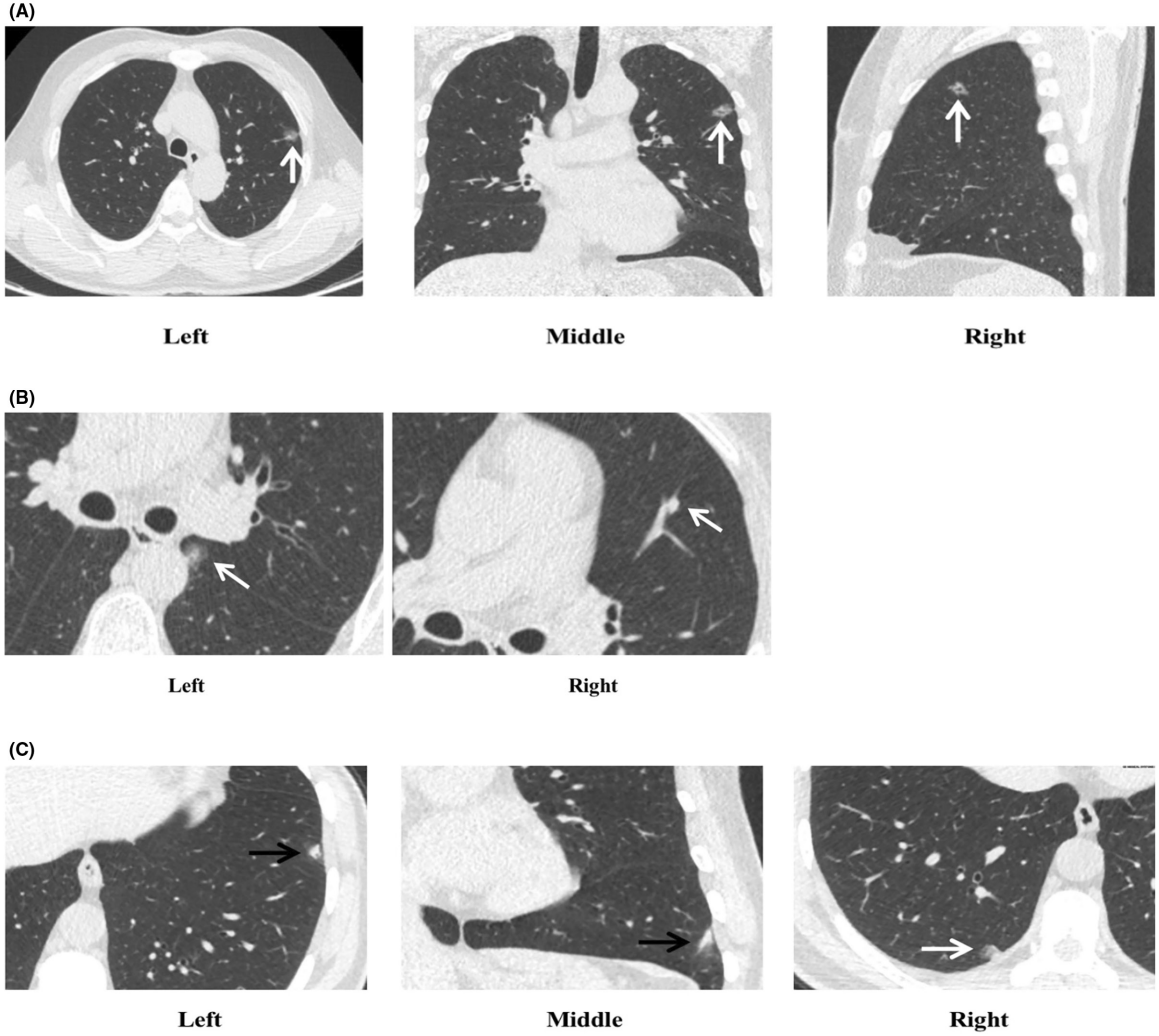
Management discrepancies stemming from same risk‐dominant nodules and different risk‐dominant nodules. (A) Depicted discrepancies in follow‐up management a rising from divergent interpretations by readers concerning the nodule type and measurements. In the first reading round, all five observers identified a subsolid nodule in the left upper lobe as the risk‐dominant nodule. Of these, three classified it as a part‐solid nodule and, due to measurement variations, recommended follow‐ups at either 3 or 6 months. Meanwhile, two observers categorized it as a ground‐glass nodule, suggesting a 12‐month follow‐up. During the second reading, with the assistance of CAD, all five observers unanimously defined the nodule as part‐solid and recommended follow‐ups at either 3 or 6 months. (B) Illustrated the discrepancy in risk‐dominant nodules due to one reader's failure to detect the nodule. The axial CT image displayed a subsolid nodule measuring 12.0 × 8.7mm (Left), located adjacent to the mediastinal pleura of the left lower lobe. In the first reading round, four observers categorized it as a part‐solid nodule: three observers recommended a 6‐month follow‐up, while one suggested a 3‐month follow‐up. Another reader, however, missed this nodule and instead chose a solid nodule in the left upper lobe as the risk‐dominant nodule, recommending a 3‐month follow‐up (Right). In the second round of reading, with the assistance of CAD, the nodule was correctly identified, with all five observers suggesting a 6‐month follow‐up. (C) Highlighted that the discrepancies in determining the risk‐dominant nodule might be attributed to variations in the nodule's definition among the observers. The axial and coronal CT scans depicted a fibrotic consolidation of approximately 10.7 mm in the left lower lobe (Left, black arrow). In the first reading round, Observers 3, 4, and 5 identified this lesion as the risk‐dominant nodule and recommended a 3‐month follow‐up. Conversely, Observers 1 and 2 pinpointed another nodule as the risk‐dominant nodule and suggested a 6‐month follow‐up (Right, white arrow). In the subsequent reading round, CAD flagged nodule (Right, white arrow) as the risk‐dominant nodule. However, despite the CAD's indication, Observers 4 and 5 mainta ined their selection consistent with the first round, whereas Observer 3 heeded the CAD's recommendation. Observers 1 and 2 stayed true to their initial selections at either 3 or 6 months.

We analyzed all 600 readings from five observers in pairs (there are 10 potential pair combinations among the five observers, 10 × 60 = 600) and found that 39.0% (234/600) of the pairs had differences in follow‐up recommendations, with major and substantial management discordance accounting for approximately 27.5% (165/600), and 4.8% (29/600), respectively. Most readings with discrepancies were associated with identical risk‐dominant nodules, accounting for 73.5% (172/234) of total readings. The proportion of readings in which the two observers allocated different follow‐up recommendations due to differences in nodule size and type was 38.0% (89/234), and 35.5% (83/234) cases, respectively (Figure 3A). In total, 18.3% (110/600) and 2.3% (14/600) of readings had major and substantial management discrepancies, respectively.

Approximately 26.5% (62/234) of the discrepancies were caused by the choice of alternative risk‐dominant nodules (Figures 3B and 3C), with major and substantial discrepancy rates of 8.0% (48/600) and 2.5% (15/600), respectively.

### 3.3 Follow‐up category agreement: Observers with CAD

In the second round, the agreement among the five observers increased, reaching substantial agreement with a Fleiss kappa value of 0.623 (0.573–0.673) (Table 2). Among all 10 pairs of readings by the five observers, the agreement increased in 9 pairs compared to the previous round. The average agreement between the pairs was higher in the second round than in the first, with a Cohen's kappa value of 0.733 (0.673–0.793), ranging between 0.646 (0.498–0.794) and 0.814 (0.697–0.930) (Table 3).

**TABLE 2:** Pairwise inter‐observer agreement in NCCN‐based follow‐up management categorization measured by weighted Cohen's Kappa.

	Reader 1	Reader 2	Reader 3	Reader 4	Reader 5
Round 1: Average, 0.609 (0.518–0.701)					
Reader 1	–	0.655 (0.503–0.807)	0.492 (0.346–0.639)	0.703 (0.557–0.849)	0.777 (0.665–0.889)
Reader 2	–	–	0.484 (0.325–0.644)	0.545 (0.368–0.722)	0.709 (0.571–0.848)
Reader 3	–	–	–	0.419 (0.264–0.574)	0.541 (0.402–0.681)
Reader 4	–	–	–	–	0.639 (0.478–0.800)
Reader 5	–	–	–	–	–
Round 2: Average, 0.733 (0.673–0.793)					
Reader 1	–	0.656 (0.507–0.806)	0.719 (0.584–0.855)	0.743 (0.614–0.873)	0.646 (0.498–0.794)
Reader 2	–	–	0.683 (0.543–0.824)	0.708 (0.579–0.836)	0.791 (0.685–0.897)
Reader 3	–	–	–	0.736 (0.598–0.875)	0.814 (0.697–0.930)
Reader 4	–	–	–	–	0.782 (0.663–0.901)
Reader 5	–	–	–	–	–

*Note*: 95% confidence intervals are shown in parentheses.

**TABLE 3:** Multirater inter‐reader agreement in NCCN‐based follow‐up management categorization measured by Fleiss kappa.

	Fleiss kappa (95% CI)	Group	*n* (%)	Fleiss kappa (95% CI)	Group	*n* (%)	Fleiss kappa (95% CI)
Round 1 (without CAD) (*n* = 60)	0.437 (0.388–0.487)	Nodule selection‐disagreed	*n* = 24	0.367 (0.288–0.446)			
		Nodule selection‐agreed	*n* = 36	0.484 (0.420–0.549)	Nodule type‐disagreed	*n* = 19	0.159 (0.052–0.265)
					Nodule type‐agreed	*n* = 17	0.745 (0.654–0.836)
Round 2 (with CAD) (*n* = 60)	0.623 (0.573–0.673)	Nodule selection‐disagreed	*n* = 21	0.561 (0.474–0.648)			
		Nodule selection‐agreed	*n* = 39	0.652 (0.589–0.715)	Nodule type‐disagreed	*n* = 10	0.419 (0.300–0.537)
					Nodule type‐agreed	*n* = 29	0.733 (0.658–0.809)

For the different risk‐dominant nodules, the agreement in recommendation improved from 0.367 (0.288–0.446) to 0.561 (0.474–0.648) in the second round. For identical risk‐dominant nodules, the agreement in recommendation increased from 0.484 (0.420–0.549) to 0.652 (0.589–0.715). In cases where there were disagreements in nodule type for identical risk‐dominant nodules, with the assistance of CAD, the agreement increased from 0.159 (0.052–0.265) to 0.419 (0.300–0.537).

In the second round, 26.2% (157/600) of follow‐up recommendation discrepancies were found in all paired 600 readings (10 × 60 = 600). Of these discrepancies, major and substantial management inconsistencies accounted for approximately 15.8% (95/600), and 1.5% (9/600), respectively.

Most readings with discrepancies were related to the same risk‐dominant nodules, accounting for 72.0% (113/157). Whereas, in 26.1% (41/157) of readings had different follow‐up recommendations due to different nodule types. Additionally, 45.9% (72/157) of patients had different follow‐up recommendations based on differences in nodule size. Major and substantial management discrepancies were observed in 9.2% (55/600) and 0.3% (2/600) of the cases, respectively.

The divergences resulting from the selection of different risk‐dominant nodules were 28.0 (44/157), with major and substantial disparities accounting for 6.7% (40/600) and 1.2% (7/600), respectively.

### 3.4 Follow‐up category changes after CAD application

**FIGURE 4:**
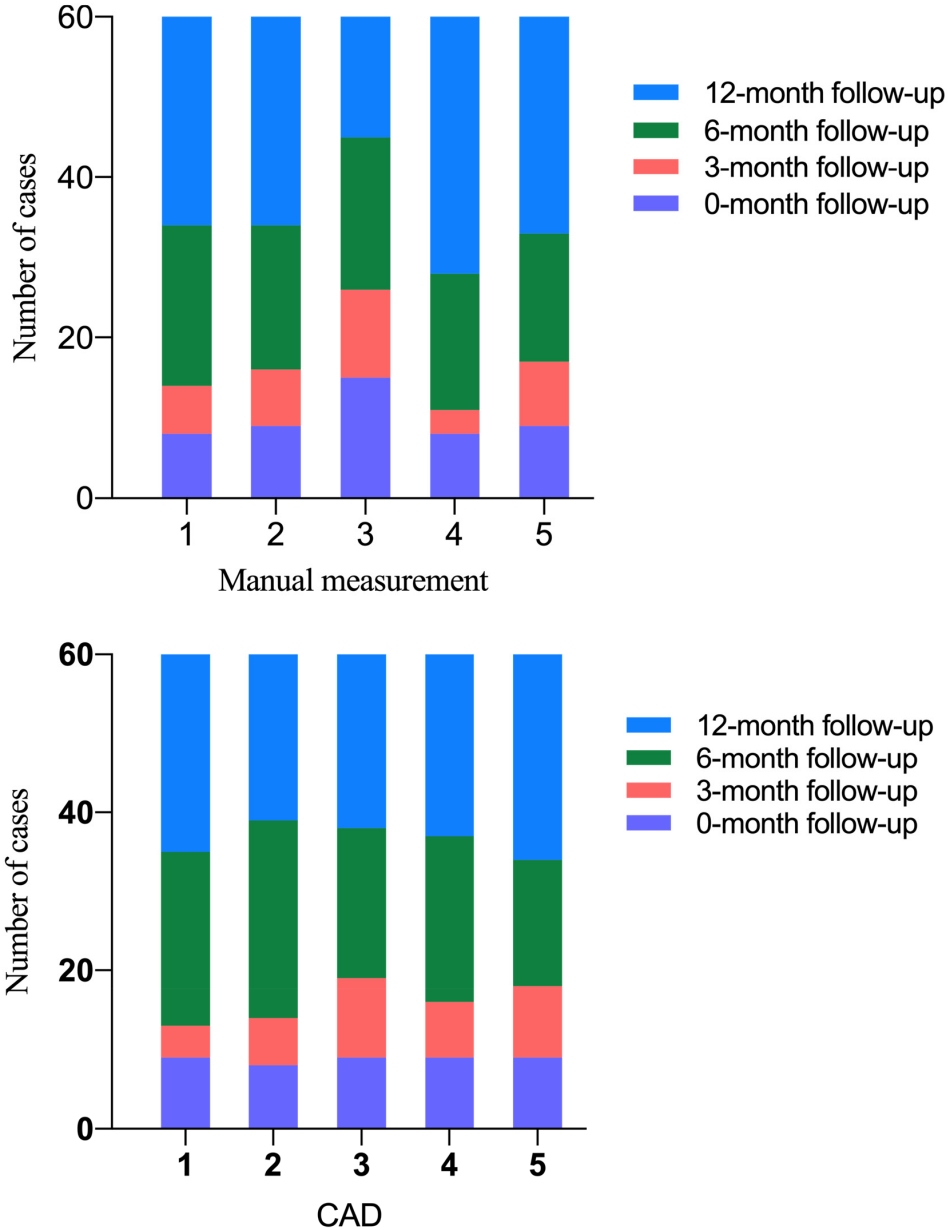
Distribution of follow‐up management classifications of all the cases (*n* = 60) among five observers when manual measurement and when using the CAD system.

We analyzed the changes in follow‐up recommendations from the five observers across two reading rounds. Among the five observers, 58.3% (35/60) to 81.7% (49/60) of cases remained unchanged across the two reading rounds. In the second reading session, follow‐up management strategies were upgraded by the observers in an average of 13.3% of cases, whereas 15.0% of cases were more likely to be downgraded. Most alterations were observed between adjacent categories relative to the first round (Table 4 and Figure 4).

**TABLE 4:** Changes to NCCN‐based follow‐up management categorization when CAD results were added.

	No change	Upstaging of NCCN categories						Downstaging of NCCN categories					
		12–6	12–3	12–1	6–3	6–1	3–1	6–12	3–12	1–12	3–6	1–6	1–3
Reader 1	46	4	0	0	2	0	2	3	0	0	2	1	0
Reader 2	49	5	1	0	0	0	0	1	0	0	3	0	1
Reader 3	35	3	1	0	0	0	0	9	1	1	5	1	4
Reader 4	43	6	2	2	2	0	1	1	0	0	1	0	2
Reader 5	42	4	2	0	2	0	1	5	0	0	3	0	1

*Note*: Data are numbers of cases.

In the first round, among 29 cancer cases (23 part‐solid nodules and 6 ground‐glass nodules), the sensitivity of the observers ranged between 62.1% (18/29) and 82.8% (24/29), with an average of 68.3%. In the second round, after CAD assistance, four observers showed increased sensitivity compared to the first round, with an average sensitivity of 73.8%, ranging between 69.0% (20/29) and 79.3% (23/29). However, the pooled sensitivity did not differ significantly (*p* > 0.05). After excluding the six ground‐glass nodules, leaving only 23 part‐solid nodules with lung cancer, we conducted a subgroup analysis. With CAD assistance, the average sensitivity was significantly improved compared to manual measurements (average, 82.6% vs. 92.2%; *p* < 0.05) (Tables 5 and 6).

**TABLE 5:** NCCN‐based follow‐up management categorization changes in 29 cancer cases.

NCCN category	CAD	Reader 1		Reader 2		Reader 3		Reader 4		Reader 5	
		Round 1	Round 2	Round 1	Round 2	Round 1	Round 2	Round 1	Round 2	Round 1	Round 2
1		6	6	6	5	8	6	6	6	6	6
3		3	1	3	3	5	3	2	4	4	3
6		10	13	9	13	11	14	10	13	10	11
12		10	9	11	8	5	6	11	6	9	9

*Note*: Data are numbers of cases.

**TABLE 6:** Comparison of observer sensitivity between two rounds in 29 cancer cases.

		Round 1			Round 2			*p*‐value*
		Sensitivity (%)	95% CI		Sensitivity (%)	95% CI		
Reader	1	65.5 (19/29)	45.7	82.1	69.0 (20/29)	49.2	84.7	1.000
	2	62.1 (18/29)	42.3	79.3	72.4 (21/29)	52.8	87.3	0.250
	3	82.8 (24/29)	64.2	94.2	79.3 (23/29)	60.3	92.0	1.000
	4	62.1 (18/29)	42.3	79.3	79.3 (23/29)	60.3	92.0	0.063
	5	69.0 (20/29)	49.2	84.7	69. 0(20/29)	49.2	84.7	1.000
	Total	68.3	60.0	75.7	73.8	65.8	80.7	0.533
		82.6			92.2			0.041

Abbreviation: CI, confidence interval.

* McNemar's test for the comparison within each reader and Logit model with generalized estimating equations in pooled data.

Among all possible 290 pairs of lung cancer case readings between the five observers (calculation as previous approach, 10 × 29 = 290), the use of CAD resulted in a reduction of approximately two‐thirds of substantial management discrepancies (differences in follow‐up time between 1/3 and 12 months), decreasing from 11 to four cases.

Regarding patient management, the observers determined a mean follow‐up period of 7.4 months in the first round and 7.3 months in the second round. Although the observers provided a slightly shorter average follow‐up interval for the second reading, the change was minimal, with an average reduction of 0.1 months.

## 4 DISCUSSION

CAD application in CT lung cancer screening is typically believed to reduce missed diagnoses and improve work efficiency. However, there has been limited research on the impact of CAD on inter‐observer agreement in terms of lung nodule management strategies. In our study, after CAD implementation, we noted a marked enhancement in agreement among observers, as evidenced by the increase in the Fleiss kappa value from 0.437 (0.388–0.487) to 0.623 (0.573–0.673).

Accurate and consistent management of nodules is imperative for effective screening of lung cancer. Among the 600 paired measurements, the average Cohen's kappa value for the manual measurements was 0.609 (0.518–0.701), ranging between 0.484 (0.325–0.644) and 0.777 (0.665–0.889). Agreement improved after using CAD, with an average Cohen's kappa value of 0.733 (0.673–0.793), ranging between 0.646 (0.498–0.794) and 0.814 (0.697–0.930). The proportion of patients with inconsistent follow‐up management decreased by 12.8% (39% vs. 26.2%) after CAD application. Major and substantial management discrepancies reduced by 11.7% (27.5% vs. 15.8%), and 3.3% (4.8% vs. 1.5%), respectively. With the introduction of CAD, we observed improved inter‐observer agreement caused by different risk‐dominant nodules, with the Fleiss kappa increasing from 0.367 (0.288–0.446) to 0.561 (0.474–0.648). However, the proportion of inconsistencies resulting from the choice of alternative risk‐dominant nodules increased slightly (26.5% vs. 28.0%). A possible explanation is that CAD may provide more nodules of similar size within the same category, potentially increasing discrepancies in the selection of risk‐dominant nodules, leading to divergences in management decisions. To address this limitation, CAD should facilitate a more detailed and in‐depth characterization of lung nodules to provide radiologists with nodule information of higher clinical relevance. The deep learning‐based CAD system developed by Trajanovski et al. integrates features of lung nodules and their surrounding background, enhancing the accuracy of nodule classification and outperforming the PanCan model in large datasets.^19^ Additionally, discrepancies in nodule definition and perception among different observers, as well as missed diagnoses by CAD, can potentially lead to discrepancies in management.

One advantage of CAD in enhancing the agreement of follow‐up management strategies is the automated identification of nodule type. In our study, compared with manual measurements, CAD significantly reduced the proportion of inconsistencies caused by discrepancies in nodule type classification (from 13.8% [83/600] to 6.8% [41/600]; *p* < 0.001). Although CAD also offers automated measurements, its impact on managing discrepancies arising from variations in nodule size measurements was limited (from 14.8% [89/600] to 12.0% [72/600]; *p* > 0.05). A possible reason for this is that the classifications in the NCCN guidelines have narrow thresholds between adjacent categories. For instance, a subsolid nodule with a solid component measuring 5.3 mm is recommended for a 12‐month follow‐up, while one measuring 6.3 mm falls into the 6‐month follow‐up category, with a mere difference of 1 mm between them. Thus, the clinical implications of such inaccuracies become most evident when the measurements approach these decision thresholds, thereby affecting the agreement of management strategies.

Our study revealed that CAD assistance significantly improved agreement among resident physicians. All three sets of paired measurements across the resident physicians showed varying degrees of enhancement (from 0.417, 0.541, and 0.639 to 0.736, 0.814, and 0.782, respectively). However, the agreement of enhancement for chest radiologists was minimal (increasing from 0.655 to 0.656). Our findings emphasize the variation in follow‐up management assessment with CAD assistance based on diagnostic experience, indicating that younger physicians may benefit more from CAD to ensure agreement in lung cancer screening results.

Another advantage of CAD is its capacity to reduce substantial management discrepancies among lung cancer cases. With the use of CAD, substantial management disagreements among the 29 lung cancer cases decreased by 63.6% (from 11 to 4 cases). Additionally, in lung cancer cases that presented as part‐solid nodules, the average sensitivity with CAD assistance was notably higher than that with manual measurement (82.6% vs. 92.2%; *p* < 0.05). Thus, CAD offers a more consistent and accurate approach in the management and detection of lung cancer. Specifically, it helps in minimizing the discrepancies in lung cancer case management, especially in cases presenting with part‐solid nodules.

Although CAD systems have demonstrated potential in enhancing inter‐observer consistency and increasing the sensitivity of detecting lung cancer cases presenting as part‐solid nodules, overdiagnosis remains a serious concern that must be addressed with diligence. Liu et al. suggest that employing varying growth thresholds to assess the growth patterns of subsolid nodules may more effectively discriminate between high‐risk subsolid nodules and indolent ones. They propose developing personalized predictive models for different growth trends of subsolid nodules to optimize management strategies and maintain a balance between the benefits and drawbacks of lung cancer screening programs.^20^ More effective lung cancer risk stratification should be realized to enhance screening outcomes in non‐smoking populations, thereby reducing overdiagnosis among those at lower risk.^21^ Meanwhile, while the high sensitivity of CAD systems has enhanced the detection of nodules, it may also lead to the over‐management of minor ground‐glass nodules, thus raising concerns about potential overtreatment. Such concerns are understandable. However, in our study, the management strategies for nodules were based on risk‐dominant nodules, and these additional minor ground‐glass nodules detected by CAD were unlikely to affect the patients' follow‐up strategies significantly.

Nonetheless, our study had several limitations. First, unlike the randomly selected CT datasets, the kappa values in this study were based on a specific dataset that evenly included all nodule categories. However, in a real‐world setting, nodules detected during screenings are often benign, predominantly requiring 6–12 months of follow‐up. Hence, evaluations should be based on a specific context rather than sweeping generalizations. Second, the sample size was relatively limited, and an expansion of the sample is necessary to enhance the generalizability of our findings. Moreover, the NCCN guidelines recommend a 1 mm scan slice thickness for LDCT. However, due to equipment variations in our study, we used slice thicknesses of 1 mm and 1.25 mm. Whether this affects the study outcomes warrants further investigation.

## 5 CONCLUSIONS

The utilization of CAD bolsters agreement in management strategies for subsolid nodules among observers, diminishes substantive management disagreements, and concurrently elevates the average sensitivity in detecting lung cancer cases presenting as part‐solid nodules.

## ETHICS STATEMENT

The Ethics Committee of Cancer Hospital, Chinese Academy of Medical Sciences approved this retrospective study and waived the need for written informed consent.

## AUTHOR CONTRIBUTIONS

**Wu Quanyang:** Data curation (lead); formal analysis (equal); investigation (lead); methodology (lead); project administration (lead); software (equal); validation (equal); visualization (equal); writing – original draft (equal). **Zhou Lina:** Formal analysis (equal); investigation (equal); writing – review and editing (equal). **Huang Yao:** Writing – review and editing (equal). **Wang Jiawei:** Writing – review and editing (equal). **Tang Wei:** Writing – review and editing (equal). **Qi Linlin:** Conceptualization (equal); formal analysis (equal). **Zhang Zewei:** Conceptualization (equal); writing – review and editing (equal). **Hou Donghui:** Conceptualization (equal). **Li Hongjia:** Conceptualization (equal); data curation (equal); investigation (equal). **Chen Shuluan:** Data curation (equal); investigation (equal). **Zhang Jiaxing:** Data curation (equal); investigation (equal). **Zhao Shijun:** Funding acquisition (supporting); methodology (lead); project administration (equal); supervision (equal); writing – review and editing (equal).

## FUNDING INFORMATION

This study was supported by the National Key Research and Development Program of China (Grant no. 2020AAA0109500) and the Chinese Academy of Medical Sciences Innovation Fund for Medical Sciences (No.2021‐I2M‐C&T‐B‐063).

## CONFLICT OF INTEREST STATEMENT

The authors have no relevant financial or non‐financial interests to disclose.

## Supporting information


Data S1.

## Data Availability

The datasets used in this study can be obtained from the corresponding author upon a reasonable request.
