# Supplementary material for: Application of computer‐aided detection for NCCN‐based follow‐up recommendation in subsolid nodules: Effect on inter‐observer agreement
Source: Cancer Med. 2024 Feb 1;13(2):e6967. doi: 10.1002/cam4.6967 (PMC10832308; doi:10.1002/cam4.6967)
Supplement: Supplementary file 1 — Data S1. [file CAM4-13-e6967-s001.pdf]

National Comprehensive Cancer Network®

NCCN Clinical Practice Guidelines in Oncology (NCCN Guidelines®)

Lung Cancer Screening

Version 1.2024 — July 19, 2023

NCCN.org

NCCN Guidelines for Patients® available at www.nccn.org/patients

Continue

Version 1.2024, 07/19/2023 © 2023 National Comprehensive Cancer Network® (NCCN®), All rights reserved. NCCN Guidelines® and this illustration may not be reproduced in any form without the express written permission of NCCN.

Printed by wu quanyang on 8/10/2023 11:32:02 AM. For personal use only. Not approved for distribution. Copyright © 2023 National Comprehensive Cancer Network, Inc., All Rights Reserved.

EVALUATION OF SCREENING FINDINGS / FOLLOW-UP OF SCREENING FINDINGS

Part-solid nodule on initial screening LDCT[o,p,q,r,dd]

- **<6 mm[t,cc]** → Annual screening LDCT[s] until patient is no longer a candidate for definitive treatment[n,q]
- **≥6 mm with solid component <6 mm[t,cc]** → LDCT in 6 mo[n] → See Evaluation (LCS-9)
- **≥6 mm with solid component ≥6 mm to <8 mm[t,cc]** → LDCT in 3 mo[n] or Consider FDG-PET/CT[u]
  - LDCT in 3 mo[n] → See Evaluation (LCS-9)
  - Consider FDG-PET/CT[u]:
    - Low suspicion of lung cancer[v] → LDCT in 3 mo[n] → See Evaluation (LCS-9)
    - High suspicion of lung cancer[v] → Biopsy[w,x,y] or Surgical excision[y,aa,bb]
      - No cancer[x] → Annual screening LDCT[s] until patient is no longer a candidate for definitive treatment[n,q]
      - Cancer confirmed → See NCCN Guidelines for Non-Small Cell Lung Cancer
- **Solid component ≥8 mm[t,cc]** → Chest CT + contrast and/or FDG-PET/CT[u]
  - Low suspicion of lung cancer[v] → LDCT in 3 mo[n] → See Evaluation (LCS-9)
  - High suspicion of lung cancer[v] → Biopsy[w,x,y] or Surgical excision[y,aa,bb]
    - No cancer[x] → Annual screening LDCT[s] until patient is no longer a candidate for definitive treatment[n,q]
    - Cancer confirmed → See NCCN Guidelines for Non-Small Cell Lung Cancer

Footnotes

Note: All recommendations are category 2A unless otherwise indicated.
Clinical Trials: NCCN believes that the best management of any patient with cancer is in a clinical trial. Participation in clinical trials is especially encouraged.

Version 1.2024, 07/19/2023 © 2023 National Comprehensive Cancer Network® (NCCN®), All rights reserved. NCCN Guidelines® and this illustration may not be reproduced in any form without the express written permission of NCCN.

Printed by wu quanyang on 8/10/2023 11:32:02 AM. For personal use only. Not approved for distribution. Copyright © 2023 National Comprehensive Cancer Network, Inc., All Rights Reserved.

EVALUATION OF SCREENING FINDINGS

FOLLOW-UP OF SCREENING FINDINGS

Nonsolid nodule on initial screening LDCT[o,p,r,dd,ee]

- **<20 mm[t]** → **Annual screening LDCT[s] until patient is no longer a candidate for definitive treatment[n,q]**
- **≥20 mm[t]** → **LDCT in 6 mo[n]** → **See Evaluation (LCS-10)**

[n] All screening and follow-up chest CT scans should use a $CTDI_{vol}$ threshold of 3 mGy or less for a patient of average size, unless evaluating mediastinal abnormalities or lymph nodes, where standard-dose CT with IV contrast might be appropriate (LCS-A). Parameters should be adjusted for patients of smaller or larger size. There should be a systematic process for appropriate follow-up. See ACR-STR Practice Parameter for the Performance and Reporting of Lung Cancer Screening Thoracic Computed Tomography (CT).

[o] The NCCN Guidelines for Lung Cancer Screening are harmonized with Lung-RADS with rounding of mean measurement to the nearest whole number (mm).

[p] Without benign pattern of calcification, fat in nodule suggestive of hamartoma, or features suggesting inflammatory etiology. When multiple nodules or other findings are present that suggest occult infection or inflammation is a possibility, suggest follow-up LDCT in 1–3 months.

[q] There is uncertainty about the appropriate duration of screening and the age at which screening is no longer appropriate.

[r] A nodule is a rounded opacity, measuring up to 3 cm in diameter. A solid nodule has a homogeneous soft-tissue attenuation, a nonsolid nodule (also known as a ground-glass nodule) has hazy increased attenuation that does not obliterate bronchial and vascular margins, and a part-solid nodule has elements of both solid and nonsolid nodules. Nodules should be evaluated and measured on LDCT using lung windows. The size of all nodules is underestimated when viewed on soft-tissue windows, and some nodules may not even be visible, particularly nonsolid nodules and small nodules. Bankier AA, et al. Radiology 2017;285:584-600.

[s] Ideally, the annual LDCT is performed 12 months from the initial or interval scan.

[t] Nodules should be measured on lung windows and reported as the average diameter rounded to the nearest whole number; for round nodules only a single diameter measurement is necessary. Mean diameter is the mean of the longest diameter of the nodule and its perpendicular diameter.

[dd] It is crucial that all nonsolid lesions be reviewed at thin (≤1.5 mm) slices to exclude any solid components. Any solid component in the nodule requires management of the lesion with the part-solid recommendations (LCS-9).

[ee] Lung-RADS 1.1 has increased the size of a nonsolid nodule that can continue with annual screening to <30 mm, rather than <20 mm as recommended in the previous version. The NCCN Guidelines Panel has not harmonized this portion of the Lung-RADS update, as the consensus among panel members is that baseline or new nonsolid nodules ≥20 mm should have an earlier evaluation at 6 months.

Note: All recommendations are category 2A unless otherwise indicated.
Clinical Trials: NCCN believes that the best management of any patient with cancer is in a clinical trial. Participation in clinical trials is especially encouraged.

Version 1.2024, 07/19/2023 © 2023 National Comprehensive Cancer Network® (NCCN®), All rights reserved. NCCN Guidelines® and this illustration may not be reproduced in any form without the express written permission of NCCN.
